# Evaluation of Poultry Stunning with Low Atmospheric Pressure, Carbon Dioxide or Nitrogen Using a Single Aversion Testing Paradigm

**DOI:** 10.3390/ani10081308

**Published:** 2020-07-30

**Authors:** Thomas C. Gent, Sabine Gebhardt-Henrich, Sarah-Lina Aagaard Schild, Abdulsatar Abdel Rahman, Michael J. Toscano

**Affiliations:** 1 Anaesthesiology Section, Department of Clinical Diagnostics and Services, University of Zurich, 8057 Zurich, Switzerland; 2 Center of Proper Housing for Poultry and Rabbits, Division of Animal Welfare, University of Bern, 3052 Zollikofen, Switzerland; sabine.gebhardt@vetsuisse.unibe.ch (S.G.-H.); abdelrahmanabdulsatar@gmail.com (A.A.R.); michael.toscano@vetsuisse.unibe.ch (M.J.T.); 3 Department of Biosystems and Technology, Swedish University of Agricultural Sciences (SLU), 230 53 Alnarp, Sweden; sarah.lina.schild@slu.se

**Keywords:** euthanasia, low atmospheric pressure stunning, CO_2_, N_2_, broiler breeder, poultry, aversion test

## Abstract

**Simple Summary:**

The use of gas stunning for poultry in the abattoir is considered preferable from a welfare and ethical perspective since it reduces the need for stressful handling and birds do not need to be separated from each other. Stunning with low atmospheric pressure is thought to be less stressful than the widely used carbon dioxide; however, there are no published studies directly comparing their aversiveness. Here we trained broiler breeders to indicate aversion to either carbon dioxide, low atmospheric pressure or the inert gas nitrogen, by relinquishing a food reward to seek a preferable environment. We found that exposure to carbon dioxide resulted in the rapid cessation of feeding, whereas with low atmospheric pressure and nitrogen, birds continued to eat for longer. We further found that carbon dioxide exposure resulted in more aversion behaviours, such as headshaking and gasping. These findings suggest that both low atmospheric pressure and nitrogen offer a welfare refinement to gas stunning with carbon dioxide in poultry.

**Abstract:**

Low atmospheric pressure stunning (LAPS) has been suggested for use in poultry under 4 kg in the abattoir as a more humane alternative to carbon dioxide (CO_2_). However, there are currently no studies offering a direct comparison of the aversion between methods. Here, we trained adult female broiler breeders to relinquish a food reward by moving to another area of the gas chamber in response to aversive stimuli. They were then stunned and subsequently killed using single exposure to either CO_2_, N_2_, LAPS or medical air as a control. Birds exposed to CO_2_ relinquished the food reward the quickest and exhibited gasping and headshaking more than the other groups. LAPS resulted in the quickest time to loss of posture (LOP) and birds in the N_2_ group took the longest. Birds exposed to N_2_ displayed the longest duration of ataxia of any group; however, they did not show any wing-flapping prior to LOP, unlike the LAPS and CO_2_. Collectively these data demonstrate that both LAPS and N_2_ are less aversive to poultry than CO_2_ and may offer a significant welfare refinement for poultry killed for meat production.

## 1. Introduction

Killing animals in a humane way requires effective stunning before killing methods are applied [1]. The order and application of these processes is relevant for both the euthanasia of sick animals and slaughter for meat production. Ideally, animals are rendered unconscious without awareness and without prior stressful procedures such as handling or transport [2]. Controlled atmosphere stunning (CAS), especially with carbon dioxide (CO_2_), is commonly used in rodents at the end of experiments [3,4], during on-farm killing required for infectious disease control [5], and in broiler poultry [6] and pigs [7] before slaughter. Gas stunning of broilers in the abattoir minimises stressful handling of awake animals prior to electrical stunning and constitutes a more welfare friendly alternative for the animals and reduced stress for involved workers [8,9]. Specifically, fixed-voltage electro-bath stunning raises specific welfare concerns as painful pressure on the distal tarsometatarsus is often required during shackling [10] to ensure sufficient electrical contact and minimise the natural variability of electrical resistance of individual birds. Furthermore, individuals with higher intrinsic resistance receive a lower current in fixed-voltage systems, which may render the stunning ineffective where birds function as multiple parallel resistors [10]. 

Upon entering the body, CO_2_ dissociates to cause intra- and extracellular acidosis, which results in a loss of consciousness, followed by cardiac arrest, as demonstrated in pigs [11]. However, prior to loss of consciousness, acidosis in mucous membranes activates nociceptors in rats [12] and poultry [13,14], which may give rise to pain, as experienced by humans [15,16]. CO_2_ has been demonstrated to be aversive to chickens and induces suffering and pain incompatible with the goal of maximising animal welfare [17,18]. For that reason, low atmospheric pressure stunning (LAPS) has been investigated as a more humane alternative to gas stunning [19,20,21]. During LAPS, the partial pressure of oxygen is gradually reduced to result in hypobaric hypoxia, leading to unconsciousness and then death [21]. Studies using behavioural observations combined with neurophysiology and cardiac responses demonstrated a progressive reduction in median spectral frequency in the EEG and early onset of slow-wave activity, which was interpreted as dose dependent sedation without excitation [21]. On the basis of these findings, it was concluded that the potential suffering of the animals during the process of stunning concluded that LAPS offered a welfare refinement for broiler birds under 4 kg [22]; however, direct comparisons of LAPS with other CAS methods have not been made for adult poultry. Furthermore, behavioural observations of LAPS in poultry have been limited to unconditioned responses, which makes the interpretation of the animals’ motivations more subjective; therefore, conditioned responses which allow a better understanding of the affective states of the animals are needed [23]. Additionally, the use of inert gases, such as nitrogen (N_2_) and argon (Ar) to induce normobaric hypoxia stunning has been investigated and demonstrated a longer onset to aversive behaviours with a lower severity [24]; however, some concerns remain over the prolonged time to loss of consciousness. Thus, the comparative refinement of alternative CAS methods of the welfare of poultry prior to slaughter remains an open question.

The aim of this study was to compare the aversion responses of poultry to CAS methods (LAPS, CO_2_, N_2_ and medical air as a control) by using a novel place preference test. We trained broiler breeders to associate a specific area of a test chamber with a positive reward (access to feed). However, dwelling in the same area could also result in exposure to minor aversive experiences (dripping water, air puffs). Animals learnt to escape the aversive stimuli by entering another chamber section at the expense of the food reward. Broiler breeders (BB) are the parental generation of broilers—i.e., chickens for meat production. Broilers have been selected for fast growth and are slaughtered long before reaching sexual maturity [25]. In order to obtain fertilized eggs from the parent generation (breeders), BB have to be subjected to severe feed restrictions, which induces long-term hunger [26,27]. These characteristics make animals easy to train for a task designed to indicate when a condition is worse than profound hunger. As secondary outcomes, we compared other aversion behaviours, including wing-flapping, headshaking and gasping [28]. We found strikingly different behavioural responses between methods but, importantly, CO_2_ resulted in rapid cessation of feeding as well as more intense headshaking and gasping compared to the others. We suggest that both LAPS and N_2_ offer welfare refinements over CO_2_ for stunning poultry for meat production.

## 2. Materials and Methods

### 2.1. Ethical Approval and Humane Endpoints

The experiment was approved by the Canton of Bern (experimental license number: BE49/19) and met all cantonal and federal legal requirements for experiments with animals in Switzerland.

Humane endpoints for this study were lameness and severe injuries. Animals showing injury or any visible lameness were immediately removed from the experiment. Visibly lame breeders were transferred to separate pens and treated with carprofen (40 mg/kg IM; Rimadyl, Zoetis). If no improvement was seen within one day of treatment the breeders were euthanised (stunned by a concussive blow to the back of the skull with a wooden club and then bled by severing the carotid arteries).

### 2.2. Animals and Housing

The study was conducted from the end of October 2019 until mid-March 2020. Eighty-five female Ross 308 broiler breeders were initially included in the study. The animals were 38 weeks of age (WOA) at the start of training and selection (see below). They had all been raised in the same building and previously participated in a behavioural study on nest choice behaviour, including regular health assessments. For experimental purposes, female birds were kept without males in deep-litter pens, measuring 11.55 × 9.7 m, which contained four nest boxes (L = 54; W = 112; H = 30 cm; see Figure S1 for complete dimensions).

Birds were fed a daily ration of dry feed (ME: 11.7 MJ/kg; crude protein: 15%; Fors Kunath, Product number: 2047.00, Burgdorf, Switzerland), given automatically at 02:00 h by a chain feeding system. Ration size was gradually decreased during the study period (170 g/bird/day at 38 WOA, 155 g/bird/day until 55 WOA, 145 g/bird/day at 56 WOA until the end of the study period).

Lighting in the home pen was set at a minimum of 5 Lux with a 16:00/08:00 h light/dark cycle and the climate in the pen was regulated by a Big Dutchman 307 pro climate control system (3900, Holland, Germany) with air temperature set at 18.0 °C (until January 2020 after which it was set at 17.5 °C).

### 2.3. Training and Test Arena

**Figure 1:**
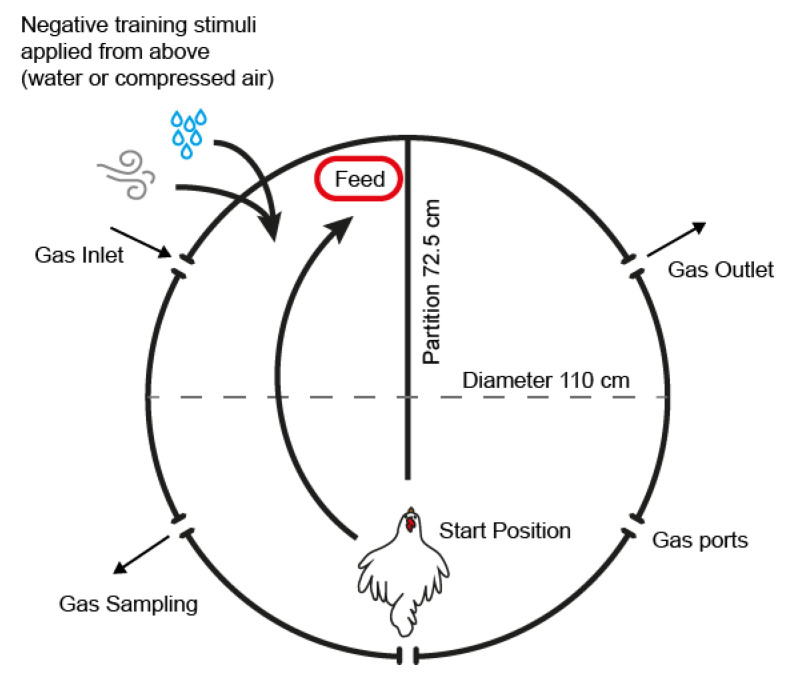
Schematic of the test arena. The start position refers to the location and orientation of the animal at the start of the training and test session. Negative stimuli for training were applied from above the animals’ heads with the chamber lid open and in the direction indicated by the arrows. Except for the hole for gas inlet, the holes for hose connection located on the sides of the arena were closed.

Training was initially performed from 41 to 49 WOA in a cylindrical wooden arena (110 cm in diameter and 62 cm in height) which was made to resemble the test arena in which final experimentation would take place (Figure 1). A wooden partition (L = 72.5 cm, H = 62 cm) was located in the middle of the arena to create two interconnected compartments. A lid made of metal wire mesh was placed on the arena to prevent the animals from escaping. The actual test arena was used for training from 50 WOA, after which the training arena was no longer used. The test arena was constructed from 15 mm stainless steel (side and flooring) with an 80 mm thick transparent acrylic lid seated on a rubber seal (Figure 1). Five ports (diameter 50 mm) were located 44 cm above ground level (40 cm distance between holes) to allow for the connection of hoses for vacuum formation, gas inflow, pressure and gas concentration measurement. A wooden floor was fitted inside the arena (1 cm thick) to which the separation wall of the same dimensions was attached.

### 2.4. Selection of Focal Animals

At 38 WOA, 50 animals were selected at random and individually marked with numbered leg bands (average weight 3745 g, from a sample of 20 birds). From then on, these animals each received 4–5 training sessions per week. The criteria for successful learning of the task was the hen approaching the feeder within 15 s after being placed in the test arena. Birds that successfully fulfilled the training criteria were marked as “suitable focal animals” and continued with training. The birds that did not consistently meet criteria in the training sessions as well as birds judged to have impeded locomotion were removed from the training paradigm. Birds which were removed, were replaced by previously unselected animals from the original pool. The cycle of recruitment for replacement continued until the training group size reached a stable population of 50 suitable focal animals and was completed at 41 WOA. Due to an unintended delay in final testing, coupled to a concern about animals becoming overweight or lame, an additional 10 focal animals were selected at 43 WOA (birds less than 4200 g, with no visible lameness) This gave a total population of 60 focal animals which met the training requirements. This number allowed for a 30% drop-out rate due to mortality, excessive weight gain, lameness, other illness or failure to feed during the final test.

### 2.5. Training Sessions

Immediately prior to training, all 60 focal animals were transferred to a waiting pen located adjacent to the home pen between 08:00 and 09:00. The metal wire mesh sides of the waiting pen allowed the focal animals to see, hear and smell birds still in the home pen. Animals had ad libitum access to water (five drinking nipples, 20 cm distance between nipples, height 48 cm) within the waiting pen but no access to food. The test room was immediately adjacent to the waiting pen. The animals were trained individually and the door between the waiting pen and the test room was closed during test sessions.

Each focal animal was gently held with both hands over the wings, on both sides of the body and briefly placed on the floor in the test room. They were then lifted into the training/test arena and placed in the arena facing the end of the wooden partition (Figure 1). The food reward was located in the left compartment, whereas no reward was available in the right compartment. In order to habituate the birds to the training, each focal animal was allowed to eat for a few seconds before being returned to the home pen. Each bird was allowed a maximum of three minutes to start eating before being removed.

Basic training was conducted 4 days/week from 38 WOA, 3 days/week during from 39 to 42 WOA, 2 days/week from 43–46 WOA, and at 47 WOA the breeders were trained once, then 4 days/week at 48–50 WOA and three days/week at 51 WOA. Training was conducted twice at 52 WOA and once again three days before the final experiment at 56 WOA.

The introduction of aversive stimuli was initiated at 43 WOA. Ten seconds after the animal began eating, a negative stimulus (compressed air or sprinkled water from overhead) was applied. Animals learnt to escape the negative stimulus by firstly stopping eating and secondly moving to the other side of the arena at the expense of the access to the food reward. All birds initially first received the air puff and, in a later session, the sprinkled water. Only one negative stimulus was used per session. The criteria for successful escape from the stimulus was defined as taking ≤15 s to exit the compartment with food (and aversive stimuli). Negative association training was conducted twice at 43–44 WOA, once at 45 WOA, twice at 50 WOA, once at 51 WOA and once at 53 WOA.

### 2.6. Final Testing

Final tests were conducted in the front of the home pen. Following completion of training, animals were randomised to receive one of four treatments (CO_2_, N_2_, LAPS, or medical air) using the RAND function in Microsoft Excel (Microsoft Office 2019 run on Windows 10 on PC). Briefly, the treatments (*n* = 10 per group) were entered in column A and then 40 random numbers were generated in column B. Columns A and B were then sorted using the SORT function based on the numerical values of column B, thus randomising the order of the treatments. Animals were picked at random from the home pen based on proximity to the nesting boxes. The numbers on the leg bands were not visible during selection and the experimenter selecting the birds (SGH) had not been involved with training and was blinded to training performance. Focal animals were individually placed in the test arena at the start point and the lid closed as the animals walked towards the feed. As soon as the animals started to feed, treatment gas flow (either CO_2_, N_2_ or medical air [O_2_: 21%; N_2_: 79%], each as 100% of gas inflow) at 30% chamber volume/min (194 L/min) or vacuum pump (Sogevac SV 65B, Leybold Schweiz AG, 6312 Steinhausen, Switzerland) calibrated to reach 333 mbar in approximately 67 s and 120 mbar in 240 s, was started (Figure S2a,b). Several CAS protocols are used commercially, including gradual and multistage fill [29]. A continuous flow was selected for this study with flow rates representative of target concentrations used in commercial systems [10]. Additionally, vacuum pump rates were calibrated to the specifications previously determined by others [30]. We chose N_2_ instead of Ar because it is already present in the atmosphere and has been reported to cause loss of posture in a shorter time [24]. Exposure to treatments lasted until 60 s following complete cessation of movement, including breathing, or a maximum of 10 min. This timescale used as the typical exposure duration for CAS in poultry was 5 min [10], therefore all behaviours could be measured and any possible unintended recovery could be observed. Vacuum pressure was recorded by reading from a calibrated manometer (188986, RS Components, Germany) during the LAPS experiments. During gas treatments, CO_2_ and O_2_ concentrations were recorded digitally at 1 Hz by a calibrated gas metre (Rapidox 3100, Cambridge Sensotec, Cambridge, UK) connected to a personal computer. Behaviour was filmed using a webcam (C250, Logitech, Switzerland) on a personal computer (Microsoft Windows 10 Camera application) and stored in MP4 format. At the end of experimentation, animals were removed from the chamber and weighed immediately. Animals exposed to medical air were first rendered unconscious by a percussive blow to the back of the head. Death was then ensured by exsanguination via the carotid arteries.

### 2.7. Behavioural Scoring

Behavioural scoring was performed post hoc by one of the experimenters who was blinded to the treatment. To test aversiveness, the primary outcome was the time taken for animals to stop eating reward and move to the other side of the chamber (Video S1). During the study, it was found that very few animals moved to the opposite side of the chamber; however, there was a consistency within treatment groups to relinquish the food reward. Since broiler-breeders are food-deprived, they have a high motivation to feed and therefore this time-point was used as a primary outcome, as used previously in rodents [31,32]. Secondary outcomes were the timings of various aversion behaviours measured in one-second bins. When two behaviours were observed at the same time (e.g., ataxia and wing-flapping), the bin was scored as the most recent behaviour to be exhibited. Definitions of the behaviours are as follows:

- Gasping was defined as wide open-mouth breathing with neck extension (Videos S2 and S3).
- Headshaking was defined as rapid side-to-side movement of the head, which occurred whilst the animal was standing or walking (Video S4).
- Wing-flapping was defined by rapid movement of the wings in a motion similar to attempted flight (Video S5).
- Jumping was defined as any vertical movement from a plantar stance, resulting in both feet leaving contact with the floor.
- Ataxia was defined as uncoordinated walking with exaggerated lateral movement or as the use of wing when standing to maintain posture (Videos S6 and S7).
- Loss of posture (LOP) was defined by cessation of standing with the head resting against either the floor or wall of the chamber (Video S8).

Note that the 60 s following complete cessation of movement, including breathing before removal from the chamber was scored as “still”.

### 2.8. Statistical Analyses

Behavioural parameters were defined as above and used to form 1 s behavioural state plots by assigning a numerical category to each. Durations, frequencies and timings of behaviours were then calculated directly from the output.

Statistical tests were performed in Graphpad Prism version 8. All behavioural parameters were tested for normality using the Shapiro–Wilk test with alpha >0.05, indicating normal distribution. Treatments were then compared using one-way ANOVA with post hoc Turkey’s correction for multiple comparisons. *p*-values < 0.05 were considered statistically significant. For the temporal distribution of behaviours, frequency of occurrence was calculated in 10 s bins and totals fitted with Gaussian curves. Continuous data are presented as mean ± standard error mean (s.e.m.) and discrete parameters as median (range) unless otherwise stated.

## 3. Results

### 3.1. Animals and Training Outcomes

The number of animals who successfully learnt the position of the food reward was 65 out of 85 (76%). Following training, animals started feeding in an average time of time of 5.6 ± 9.3 s after being placed in the chamber. The two negative stimuli resulted in different times to relinquish the food rewards (water: 5.6 s ± 15.3 s; compressed air: 22.8 ± 20.0 s; *p* < 0.0001; Wilcoxon test; χ^2^_1_ = 19.3).

Forty-two animals were subsequently used for behavioural measurements with an age of 57 WOA at the time of testing. Three animals were euthanised due to lameness before final experimentation. There were no differences in average body mass between treatment groups (Figure S2c; Table 1). Two animals were excluded from final experimentation; in both cases the experiment was aborted before completion. A total of 10 animals were used in each treatment group. The first was aborted due to technical difficulties with gas delivery and the second animal did not resume feeding after closing the chamber lid and therefore gas flow could not be initiated.

Average values for behavioural parameters and summary of statistical tests between treatment groups.

### 3.2. General Behaviour Patterns

**Figure 2:**
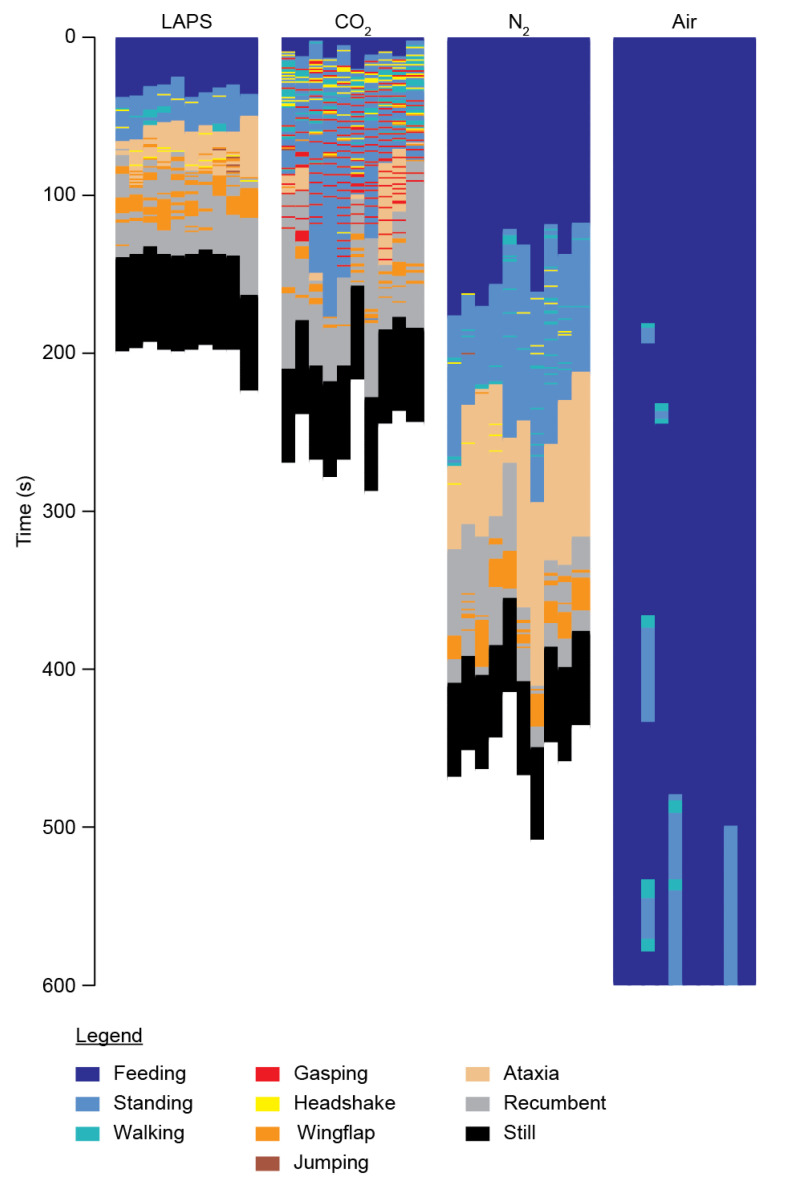
Grouped behavioural plots of all bird undergoing CAS.The graphical plots show the behaviours of the birds in 1 s bins categorised by treatment group. Colour coding for each behaviour is shown in the legend at the bottom. Note that “Still” (black) shows the 60 s observation period following cessation of all observable movement, including breathing, before the termination of experiments.

Categorical behavioural plots by treatment group over time revealed strikingly different distributions of behaviour (Figure 2), particularly in durations of feeding and the timing of aversion behaviours, such as headshaking and wing-flapping. Of particular note is the incidence of gasping (red bars) in the CO_2_ group, which was not seen in other treatment groups. Numerical averages (mean), variances and statistical values for all parameters and treatment groups are given in Table 1. 

### 3.3. Primary Aversion Behaviours

**Figure 3:**
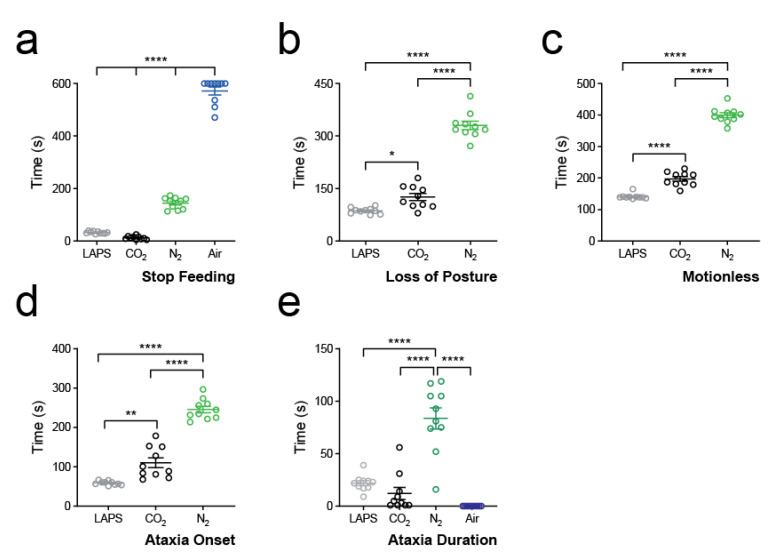
Timing of primary outcomes. (**a**) Raw data, mean ± standard error mean (s.e.m.), of time to stop feeding for all treatment groups. **** *p* < 0.0001; one-way ANOVA. (**b**) Raw data, mean ± s.e.m., of time to loss of posture (LOP) for LAPS (grey), CO_2_ (black) and N_2_ (green) treatment groups. * *p* < 0.05, **** *p* < 0.0001; one-way ANOVA. Note that LOP was not observed in any bird in the medical air group. (**c**) Raw data, mean ± s.e.m., of time to motionless for LAPS (grey), CO_2_ (black) and N_2_ (green) treatment groups. **** *p* < 0.0001; one-way ANOVA. Note that motionlessness was not observed in any bird in the air group. (**d**) Raw data, mean ± s.e.m. of time to onset of ataxia for LAPS (grey), CO_2_ (black) and N_2_ (green) treatment groups. Note that no ataxia was observed in any bird in the air group. ** *p* < 0.01, **** *p* < 0.0001; one-way ANOVA. (**e**) Raw data, mean ± s.e.m. of ataxia duration for all treatment groups. **** *p* < 0.0001.

We found that only three animals in total crossed to the opposite side of the chamber during gas exposure (CO_2_: *n* = 1; N_2_: *n* = 1; LAPS; *n* = 1). We did, however, observe a consistent time course for birds to relinquish the food reward and turn away from the food hopper. We therefore decided to quantify this as the primary aversion outcome. We found differences between all groups (*p* < 0.0001, F = 958.1; one-way ANOVA; Figure 3a). Animals exposed to CO_2_ rapidly stopped eating (12.4 ± 2.0 s) at a point equating to a concentration of 3.1 ± 0.4% (Figure S2b, right panel). Seven out of the ten birds exposed to medical air ate continuously for 10 min, giving a group average of over 9.5 min (571.9 ± 15.1 s).

Animals in LAPS reached LOP quicker than other groups (86.6 ± 2.8 s; *p* < 0.0001, F = 203.6; one-way ANOVA; Figure 3b; pressure curve see Figure S2a), whereas N_2_ animals (330.3 ± 11.9 s) took longer. We then further quantified the time to motionless as an indicator of death. We found that LAPS animals reached motionlessness faster than other groups (141.2 ± 2.7 s, *p* < 0.0001, F = 464.8; one-way ANOVA; Figure 3c) and N_2_ animals slower when compared to the other groups (399.4 ± 7.9 s).

The onset to ataxia was quickest in the LAPS group (59.0 ± 1.6 s, *p* < 0.0001, F = 125.1; one-way ANOVA; Figure 3d) and was prolonged in N_2_ animals (246.0 ± 8.2 s). Interestingly, there was no difference in duration of ataxia between LAPS (22.0 ± 2.4 s) and CO_2_ (12.2 ± 5.7 s; *p* = 0.65; Tukey’s multiple comparisons test; Figure 3e). Ataxia duration was longer in N_2_ animals (83.7 ± 10.1 s; *p* < 0.0001, F = 39.8; one-way ANOVA). We did not observe any ataxia in animals exposed to medical air.

### 3.4. Secondary Aversion Behaviours

In addition to the primary behavioural outcomes, we also observed other behaviours which are indicative of aversion and quantified them accordingly.

**Figure 4:**
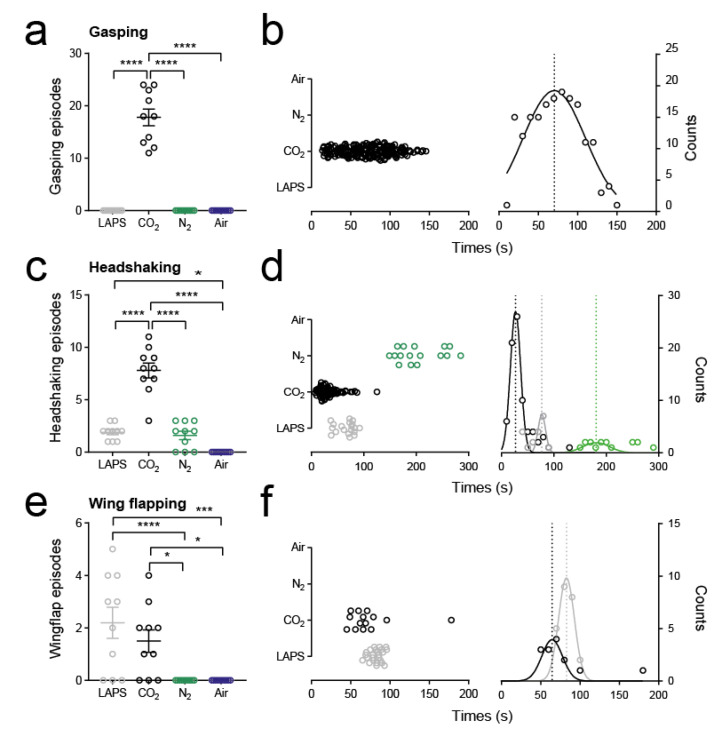
Incidence and timing of aversion behaviours. (**a**) Raw data, mean ± standard error mean (s.e.m.), of number of gasping episodes per bird categorised by treatment group. **** *p* < 0.0001; one-way ANOVA. (**b**) Left panel shows a cluster plot of all gasping episodes with respect to time from all animals. Right panel shows frequency occurrence of gasping episodes sorted into 10 s bins. The data are fit with Gaussian curves. The asymptote marks the mean of the distribution. (**c**) Raw data, mean ± s.e.m., of number of headshaking episodes per bird categorised by treatment group. * *p* < 0.05, **** *p* < 0.0001; one-way ANOVA. (**d**) Left panel shows a cluster plot of all headshaking episodes with respect to time from all animals. Right panel shows frequency occurrence of headshaking episodes sorted into 10 s bins. The data are fit with Gaussian curves. Asymptotes marking the means of the distributions for each treatment group are also shown. (**e**) Raw data, mean ± s.e.m., of number of wing-flapping episodes prior to LOP per bird categorised by treatment group. * *p* < 0.05, *** *p* < 0.001, **** *p* < 0.0001; one-way ANOVA. (**f**) Left panel shows a cluster plot of all wing-flapping episodes prior to LOP with respect to time from all animals. Right panel shows frequency occurrence of wing-flapping episodes sorted into 10 s bins. The data are fit with Gaussian curves. Asymptotes marking the means of the distributions for each treatment group are also shown.

We observed multiple gasping episodes (median 18, range 11–24; Figure 4a) by all animals exposed to CO_2_. Temporal distribution analysis of these episodes revealed that gasping was seen within the first 10 s of gas exposure and the Gaussian equation fits showed that the peak incidence of gasping occurred at around 70 s (Figure 4b). We further observed gasping episodes occurring after LOP in 50% of animals. By contrast we did not observe gasping behaviours in the other treatment groups. We did observe open mouthed breathing in LAPS and N_2_ groups; however, all observed episodes occurred after LOP. Furthermore, the mandibular angle during such open mouth breathing (5–10°; Video S9) was noticeably different from gasping (40–45°) and was not associated with the extension of the neck.

Headshaking was observed in LAPS, CO_2_ and N_2_ groups. All episodes were observed before LOP and were more frequent in the CO_2_ group (8 (3-11) episodes per animal; *p* < 0.0001, F = 64.9; one-way ANOVA; Figure 4c). Temporal distribution analysis revealed that most headshaking episodes occurred in the CO_2_ groups at the beginning of exposure and tapered off as the concentration increased (Figure 4d). In contrast, headshaking in LAPS and N_2_ groups occurred more as the animals approached LOP. We did not observe any headshaking in the medical air group, suggesting this behaviour resulted from the CAS treatment only.

We observed jumping in five animals (LAPS: *n* = 2; CO_2_: *n* = 2; N_2_: *n* = 1, Figure 2, quantification not shown). Jumps appeared qualitatively different, where some were associated with wing-flapping, some with headshaking and some with ataxia. Only one jump from a total of seven episodes was not immediately preceded by another aversion behaviour. Due to the very low incidence and heterogeneous appearance, we did not statistically analyse jumping.

Finally, we observed wing-flapping in the LAPS, CO_2_ and N_2_ groups. Visual inspection of behavioural plots revealed different temporal distributions of wing-flapping (Figure 2), notably that all episodes in the N_2_ group occurred after LOP. To assess the possible association on impaired welfare, we quantified all wing-flapping episodes which occurred prior to LOP. We found no difference in the number of episodes per animal between LAPS and CO_2_ (LAPS: 2.5 (0–5); CO_2_: 1.5 (0–4); *p* = 0.54; Tukey’s multiple comparison test; Figure 4e). Temporal distribution analysis revealed that most episodes in CO_2_ occurred during the middle of exposure, whereas in the LAPS group, episodes occurred as animals approached LOP (Figure 4f).

## 4. Discussion

### 4.1. Novelty of Methods

The current study sought to evaluate broiler breeder behavioural responses following exposure to CO_2_, N_2_, or LAPS where the animal is not intended to recover. With the exception of CO_2_ stunning, the CAS methods demonstrated here are not frequently used in the poultry industry, where electrical stunning remains the most common method. However, welfare concerns have led to a concerted search for more humane alternatives. The need for effective methods, including the use of inert gases or LAPS, which can be performed on farm, is only expected to increase in parallel with growing farm size and the need to depopulate in the event of disease outbreak. While aversion testing is common with CAS investigations [31,33,34] including poultry [6,35], we believe our testing procedure is the first to use a singular conditioned testing paradigm and test apparatus to compare LAPS to other CAS methods. Although preferences and aversions to different combinations of gases are possible within a single test apparatus [6,36], the inclusion of LAPS and the associated technical demands (i.e., a chamber of capable strength) required a custom built apparatus which the current study was able to utilise. Restricted observations within a commercial LAPS system, indicating a similar behavioural sequence (e.g., ataxia and alertness followed by LOP and convulsions; [19,30]) and onset latency (e.g., headshaking; [19]) suggest the current paradigm is relevant to commercial conditions. As the testing procedure used in this effort involved birds thoroughly acclimated to the apparatus and an unobstructed view of hen behaviour was provided for the entire exposure period, we believe our results make a novel contribution to the field.

### 4.2. General Behaviour

Examination of the behavioural profiles revealed clear differences between treatments for multiple behaviours culminating in distinctive treatment-specific patterns. For instance, the profile of LAPS and N_2_ were similar in the order and occurrence of key behaviours, though the latter involved relatively longer phases resulting in a doubling of duration until stillness. At the more granular level, N_2_ animals took twice as long to stop feeding and four times as long until the onset and duration of ataxia. In that sense, although the N_2_ and LAPS had similar sequential profiles, LAPS appeared to have a more rapid effect in comparison to N_2_ which could be taken as a benefit through reductions in cumulative stress, especially when combined with reduced averseness [37]. Whether or not increasing N_2_ gas flow rates would result in the same temporal profiles of behaviour as LAPS, as performed in the context of this study, remains to be determined. In contrast to LAPS and N_2_, birds exposed to CO_2_ had a relatively rapid cessation of feeding followed by an extended period of standing, punctuated by gasping that continued beyond 100 s. Gasping, believed to be indicative of aversive conditions and discussed in detail below, occurred only in the CO_2_ exposed animals. Headshaking, also believed to indicate aversiveness [35], occurred in all treatments (except medical air), albeit with greater frequency, earlier, and within a narrower window for CO_2_ exposed birds. Together these results suggest a more intense aversion experienced at the beginning of CAS with CO_2_.

### 4.3. Signs of Aversion

Our study was intended to focus on indicators of aversiveness and involved an extensive training regimen where the birds learned to associate the non-feeding side of the chamber as a safe and easy means to escape negative stimuli. Although all tested animals readily responded by transitioning when exposed to several negative stimuli during training, very few (*n* = 3) made the transition during CAS exposure across all treatments, therefore this behaviour was not considered a reliable measure. We are uncertain why there was a lack of the conditioned response, although we believe several factors may be responsible, including: disorientation in response to CAS, a finding supported by the occurrence of ataxia, as well as different persons conducting the tests and training. Future experiments may consider using a range of gas concentrations and delivery rates to see if similar behavioural responses occur with reductions in ataxia. Additionally, due to logistical reasons the application of negative stimulus training ended four weeks before the tests, which might have led to the extinction of the learned response [38]. Furthermore, the birds may not have been able to localise the source of the negative stimulus, unlike the direct air puffs and water. Finally, birds might have failed to generalise the negative stimuli received during training [39]. We designed our procedure with the belief that the intended results would provide a clear indication of the animals’ impression and preference, relative to a resource that was unambiguously attractive [31]—i.e., feed— as indicated by continuous feeding seen in birds exposed to medical air. We believe the concept retains merit and should be considered for future efforts, although the benefits of simpler and more comparable training and testing tasks should be evaluated.

Despite the lack of transition to the other side of the chamber, other behavioural responses suggest differences in aversiveness between CAS methods. Given the attraction of the feed, the rapid cessation of eating, most pointedly by CO_2_ exposed birds could be interpreted as signalling aversion, but also confusion, disorientation, or an innocuous shift in attention as the bird becomes aware of its changing environment. Awareness of environmental change is a vital adaptive trait for all animals which may or may not be associated with a priori aversion [40]. Later responses, most notably gasping and headshaking during CO_2_ exposure [9,18], are frequently used as indicators of aversion, although their function is not clear and may vary across conditions. Gregory et al. [41] is normally credited with suggesting that headshaking during CO_2_ exposure indicates aversion and respiratory distress as high concentrations are perceived by humans as irritant [15]. Although widely mentioned, the association of behaviours with aversion have not been validated, though the parallel comparisons offered within the current study are informative. For instance, Webster and Fletcher [9], citing work that headshaking occurred in response to novel or disturbing stimuli and was an effort to self-arouse [42], suggested headshaking may have resulted from disorientation experienced during CO_2_ exposure. Observations by others that headshaking occurred following several deep breaths [28] support the concept. Alternatively, headshaking may also occur in response to mucous membrane irritation following initial exposure to CO_2,_ for which Webster and Fletcher [9] suggested the rapidity of the response and performance over the course of exposure would be important to determine its physiological underpinnings. We did not observe headshaking during routine housing, training sessions or control experiments with medical air, thus ruling out infection as a cause. Our results do support differential mechanisms, where LAPS and N_2_, with relatively dispersed headshakes, could be interpreted as resulting from disorientation. In contrast, birds exposed to CO_2_ performed rapid and early headshakes that gradually gave way to gasping. Unfortunately, results are variable across studies using CO_2_ with headshaking occurring both before [43] and after [28] deep breaths. As an explanation for headshaking during LAPS, the behaviour was suggested to equalise pressure between the ears and oral cavity which would relieve discomfort in the head, although the overall benefit of this is not clear [19]. The parallel comparisons of the current work, ideally combined with the intended unambiguous transition to the “safe” side of the chamber, would clarify the nature of these responses and their usefulness in evaluating CAS methods.

Gasping is also widely seen as a response to aversive conditions, though its function is similarly unclear. Air hunger, with which gasping is associated [44], has been cited as the main cause of stress during CAS [1] and is defined as the negative and urgent sensation experienced by humans when holding their breath beyond a normal physiological capacity [45]. Gerritzen et al. [17,43] found that reducing CO_2_ concentration with increasing O_2_ did not eliminate gasping but did affect the intensity and duration, suggesting a moderating effect. We saw gasping only in the CO_2_ exposed birds, although other evidence of breathing compromise—i.e., open mouth breathing—was observed in the N_2_ and LAPS groups. While similar in nature, the dramatic differences regarding the mandible angle and neck extension suggest a different aetiology and function. Open mouth breathing has also been reported in LAPS-exposed birds [19] where gasping was similarly not reported. It is also possible that a clarification of the terms is needed as Gerritzen et al. [17] defined gasping as deep breathing with an open mouth and neck stretching as separate behaviours, whereas Mackie and McKeegan [19] used the same definition of gasping but “with or without neck extension”.

Wing-flapping prior to LOP could also be interpreted as a sign of aversiveness if the animals were trying to escape, though this would typically be associated with crouching or other movement preceding flight, which was not observed in the current study. Jumping did occur rarely but did not appear to be associated with wing-flapping. Wing-flapping is more likely related to anoxia and consequent loss of consciousness and myoclonic seizures [22], which is supported by the occurrence after ataxia but before LOP, most prominently in the LAPS and N_2_. The occurrence of wing-flapping is also important in terms of carcass quality as, when performed rapidly, would lead to increased lactic acid in the muscle, though no differences between treatments were observed. Regarding tissue damage, LAPS can induce dramatic pathological changes, including lesions in the calvaria, brain, heart and lungs, though it is not clear if the injuries result from decompression (and during consciousness) or recompression [30]. The current study observed bleeding from the head in LAPS exposed animals as well as prolapses likely related to the rate of decompression/recompression [19,30].

## 5. Conclusions

Our study evaluated three CAS methods within a single aversion testing paradigm that offered the ability for close behavioural observations of animals that had been habituated to the procedure. Although we were unable to collect the full breadth of aversion responses, our ability to compare CAS methods in parallel suggested very different patterns of behaviour. Most notably, episodes of gasping and headshaking in LAPS exposed animals had an earlier onset and came with sporadic bursts in the period leading to recumbency compared to CO_2_ and N_2_ exposed hens. We conclude that aversion from CO_2_ was greater than with other groups and that LAPS and N_2_ offer a welfare refinement for stunning birds in the abattoir setting. However, future works would benefit from the inclusion of physiological measurements during CAS to determine their role in the observed behaviour.

## Figures and Tables

**Table 1:** Quantification of Behavioural Parameters.

Parameter	Group	Average (Mean)	Variance (s.e.m.)	ANOVA	CO_2_	N_2_	Air
Body mass (kg)	LAPS	4.25	0.10	*p* = 0.6324; F = 0.5797	0.9172	0.7450	0.9329
	CO_2_	4.38	0.12			0.9329	0.9329
	N_2_	4.45	0.11				0.9329
	Air	4.36	0.07				
Feeding time (s)	LAPS	32.30	1.391	*p* < 0.0001; F = 958.1	<0.0001	<0.0001	<0.0001
	CO_2_	12.40	2.023			<0.0001	<0.0001
	N_2_	144.7	7.002				<0.0001
	Air	571.9	15.14				
Headshaking (no. episodes)	LAPS	1.900	0.2333	*p* < 0.0001; F = 64.88	<0.0001	0.9586	0.0159
	CO_2_	7.800	0.7118			<0.0001	<0.0001
	N_2_	1.600	0.4000				0.0534
	Air	0.000	0.000				
Headshaking (time of first episode)	LAPS	58.40	6.344	*p* < 0.0001; F = 155	0.0001	<0.0001	-
	CO_2_	14.90	1.958			<0.0001	-
	N_2_	185.6	12.46				-
	Air	-	-				
Gasping (no. episodes)	LAPS	0.000	0.000	*p* < 0.0001; F = 139.5	<0.0001	>0.9999	>0.9999
	CO_2_	18.40	1.558			<0.0001	<0.0001
	N_2_	0.000	0.000				>0.9999
	Air	0.000	0.000				
Jumping (no. episodes)	LAPS	0.4	0.3055	*p* = 0.4206; F = 0.9633	0.8480	0.6190	0.3776
	CO_2_	0.2	0.1333			0.9770	0.8480
	N_2_	0.1	0.1000				0.9770
	Air	0.0	0.000				
Wing flaps (no. episodes pre-LOP)	LAPS	2.1	0.5667	*p* = 0.0001; F = 9.04	0.6341	0.001	0.001
	CO_2_	1.5	0.4282			0.0249	0.0249
	N_2_	0.0	0.000				>0.9999
	Air	0.0	0.000				
Wing flap duration (post-LOP(s))	LAPS	16.50	1.344	*p* < 0.0001; F = 14.74	0.0016	0.3931	-
	CO_2_	7.100	1.362			<0.0001	-
	N_2_	19.70	2.251				-
	Air	-	-				
Ataxia onset (s)	LAPS	59.00	1.626	*p* < 0.0001; F = 125.1	0.0074	<0.0001	-
	CO_2_	110.6	12.41			<0.0001	-
	N_2_	246.0	8.192				-
	Air	-	-				
Ataxia duration (s)	LAPS	22.00	2.418	*p* < 0.0001; F = 39.77	0.6474	<0.0001	0.0573
	CO_2_	12.20	5.700			<0.0001	0.4714
	N_2_	83.70	10.06				<0.0001
	Air	0.0	0.000				
Time to LOM (s)	LAPS	141.2	2.724	*p* < 0.0001; F = 464.8	<0.0001	<0.0001	-
	CO_2_	197.4	7.011			<0.0001	-
	N_2_	399.4	7.901				-
	Air	-	-				

## Supplementary Materials

The following are available online at https://www.mdpi.com/2076-2615/10/8/1308/s1, Figure S1: Schematic of home pen, Figure S2: Gas concentration and pressure curves, Video S1: Cessation of feeding, Video S2: Gasping—overhead view, Video S3: Gasping—side view, Video S4: Headshaking, Video S5: Wingflapping, Video S6: Ataxia, Video S7: Ataxia, Video S8: Loss of posture, Video S9: Open mouth breathing.
