# Supplementary material for: Evaluation of Poultry Stunning with Low Atmospheric Pressure, Carbon Dioxide or Nitrogen Using a Single Aversion Testing Paradigm
Source: Animals (Basel). 2020 Jul 30;10(8):1308. doi: 10.3390/ani10081308 (PMC7459835; doi:10.3390/ani10081308)
Supplement: Supplementary file 1 [file animals-10-01308-s001.zip › Supplementary.docx]

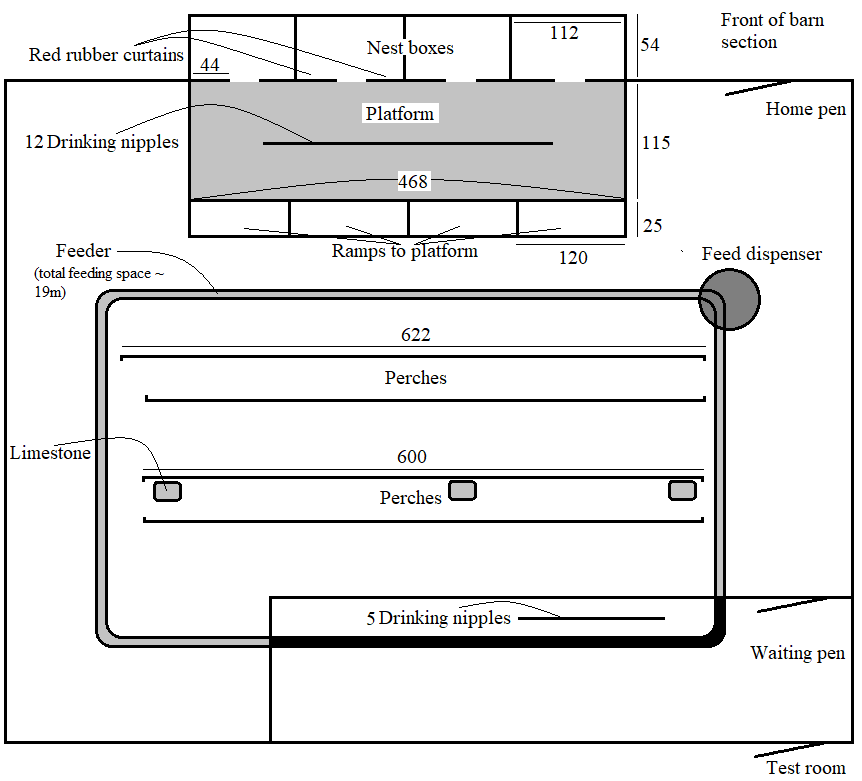


# Supplementary Figure 1: Schematic of the home pen.

‘Waiting pen’ refers to the pen where the animals were placed on the morning of training days before moving to the test room. In the waiting pen the animals had access to nipple drinkers but the feeder was closed (as indicated in black in the figure) and thus no feed was available. All measures are in centimetres unless stated otherwise in the figure.

The nest boxes were located at a height of 72 cm above the ground and the entrance to each box was partly covered by two red rubber curtains (44 x 30 cm). A platform of white plastic slats (468 x 115 cm) was located in front of the nest boxes and sloped slightly downward from the nest boxes towards the floor (from a height of 58 cm to a height of 46 cm). Four ramps made of metal mesh (25 cm wide and 120 cm long), allowed chickens to access to the platform. Nipple drinkers (12 nipples, 24 cm distance between nipples) were placed on the platform 48 cm above the platform surface. In the middle of the pen, two pairs (36 cm distance within a pair) of standard round plastic-coated metal (D = 3.5 cm) perches were located (one was 622 cm long and the remaining three 600 cm long) at a distance of 83 cm between the pairs and a height of 50 cm. Three blocks of limestone (~1.5kg/piece) were placed on the floor underneath the perches. The chain feeding system measured (29 cm above ground level) with ~19 m of feeding space

#
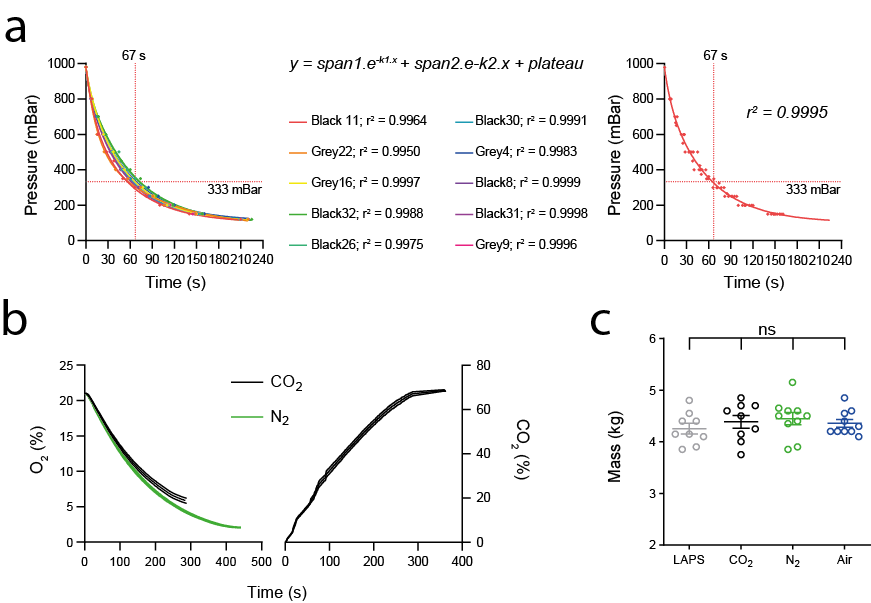


# Supplementary Figure 2

**a.** Left panel shows pressure plots with respect to time for individual experiments for all birds undergoing LAPS exposures. Data are fitted with two-phase exponential decay functions. Asymptotes at 67 s (x-axis) and 333 mbkpar (y-axis) indicate first target pressure for the LAPS procedure. Centre panel shows the model equation for the two-phase exponential curve fit. Below are the colour codes for the individual data and curve fits for each bird, labelled as bird identity within the flock. Right panel shows all pressure:time data points from all birds and a single two-phase exponential decay curve fitted to all points. Target pressure and time asymptotes are also shown. **b.** Left panel shows mean oxygen concentration with respect to time for all experiments in CO_2_ (black) and N_2_ (green) treatment groups. S.E.M. boundaries are also shown. Note that curves stop in correspondence to the end of experiments. Right panel shows CO_2_ concentrations with respect to time for all CO_2_ experiments. s.e.m. boundaries are also shown. **c.** Histogram shows raw data, mean ± s.e.m. of mass of all birds, categorised by treatment group. No significant differences were found between groups (P = 0.63; F = 0.58; one way ANOVA; ns = not significant).
